# A Comparison of Different Approaches to Estimate Small-Scale Spatial Variation in Outdoor NO_2_ Concentrations

**DOI:** 10.1289/ehp.0901818

**Published:** 2010-12-30

**Authors:** Marieke B. Dijkema, Ulrike Gehring, Rob T. van Strien, Saskia C. van der Zee, Paul Fischer, Gerard Hoek, Bert Brunekreef

**Affiliations:** 1 Department of Environmental Health, Municipal Health Service Amsterdam (GGD), Amsterdam, the Netherlands; 2 Institute for Risk Assessment Sciences (IRAS), Utrecht University, Utrecht, the Netherlands; 3 Centre for Environmental Health Research, National Institute for Public Health and the Environment (RIVM), Bilthoven, the Netherlands; 4 Julius Center for Health Sciences and Primary Care, University Medical Center Utrecht, Utrecht, the Netherlands

**Keywords:** air pollution, dispersion, land-use regression, NO_2_, traffic

## Abstract

**Background:**

In epidemiological studies, small-scale spatial variation in air quality is estimated using land-use regression (LUR) and dispersion models. An important issue of exposure modeling is the predictive performance of the model at unmeasured locations.

**Objective:**

In this study, we aimed to evaluate the performance of two LUR models (large area and city specific) and a dispersion model in estimating small-scale variations in nitrogen dioxide (NO_2_) concentrations.

**Methods:**

Two LUR models were developed based on independent NO_2_ monitoring campaigns performed in Amsterdam and in a larger area including Amsterdam, the Netherlands, in 2006 and 2007, respectively. The measurement data of the other campaign were used to evaluate each model. Predictions from both LUR models and the calculation of air pollution from road traffic (CAR) dispersion model were compared against NO_2_ measurements obtained from Amsterdam.

**Results and conclusion:**

The large-area and the city-specific LUR models provided good predictions of NO_2_ concentrations [percentage of explained variation (*R*^2^) = 87% and 72%, respectively]. The models explained less variability of the concentrations in the other sampling campaign, probably related to differences in site selection, and illustrated the need to select sampling sites representative of the locations to which the model will be applied. More complete traffic information contributed more to a better model fit than did detailed land-use data. Dispersion-model estimates for NO_2_ concentrations were within the range of both LUR estimates.

Many epidemiological studies have shown that air pollution is associated with health effects such as cardiopulmonary morbidity and mortality (Brunekreef and Holgate 2002; Pope and Dockery 2006). Currently, the land-use regression (LUR) method (Briggs et al. 1997) is being used increasingly for estimating small-scale variations in air pollution concentrations in European and North American epidemiological studies (e.g., Hoek et al. 2008; Smith et al. 2006). The quality of LUR-based exposure estimation of outdoor air pollution concentrations relies largely on coverage and quality of specific monitoring campaigns and the geographic data to support them. The information that can be extracted from land-use maps depends on the resolution of these maps, which is often limited. Another common limitation is that digital geographic traffic information of traffic is usually not readily available and must be collected from local and national authorities and linked to digital road maps.

Most LUR studies report good performance of prediction models, expressed as the explained variation (*R*^2^) (Hoek et al. 2008). Validation is often performed by internal leave-one-out cross-validation from the database used for developing the model. An independent data set for model validation is not often available.

Dispersion modeling is another method to estimate small-scale variations in air pollution concentrations. In the Netherlands, the CAR (calculation of air pollution from road traffic ) dispersion model (Eerens et al. 1993) is widely used for the purpose of air-quality management and regulation. Few comparisons have been made between dispersion and LUR models (Briggs et al. 2000; Cyrys et al. 2005; Marshall et al. 2008).

For the purpose of this study, we had two independent data sets of nitrogen dioxide (NO_2_) measurements in the city of Amsterdam that allowed us to evaluate the performance of the LUR models in predicting concentrations from the data set not used for model development. The aims of our study were to evaluate the value of complete traffic data that are not generally available and high-resolution land-use data for improving LUR model performance, to evaluate the performance of two LUR models with independent sets of NO_2_ measurements, and to compare the ability of the CAR dispersion model and two LUR models to estimate small-scale variations in NO_2_ concentrations.

## Methods

### Study areas

The study area for the large-area LUR is situated in the northwestern part (6,000 km^2^) of the Netherlands [see Supplemental Material, Figure 1 (doi:10.1289/ehp.0901818)]. It includes rural, suburban, and urban areas that include major cities such as Amsterdam and Rotterdam. With 4.2 million inhabitants in almost 2 million households, this part of the Netherlands is densely populated and has a dense (tight) network of roads. The study area for the city-specific LUR model consists of the greater city of Amsterdam (1 million inhabitants, 170 km^2^) (see Supplemental Material, Figure 1).

### Air quality

We conducted two independent NO_2_-monitoring campaigns. The campaign for the large-area model took place in 2007 using Ogawa passive samplers (Ogawa & Company, Pompano Beach, Florida, USA). A total of 60 samplers were distributed in traffic-dominated urban sites (*n* = 18), nontraffic urban sites (*n* = 34), and rural sites (*n* = 8). Eight additional samplers were located at rural sites outside the study area to minimize border effects when calculating background concentrations (Beelen et al. 2007). All samplers were located on the façade of residential buildings and away from local sources (e.g., chimneys) other than traffic. We performed 1-week monitoring (7 days ± 3 hr, all starting on the same day) in all four seasons (January, April, June, and October). Sampling and analysis were carried out as described earlier (Van Roosbroeck et al. 2006).

For the city-specific model, we used 2006 data from a routinely performed passive NO_2_ monitoring program with Palmes tubes (Palmes et al. 1976) in Amsterdam (van der Zee and van Wijnen 2004). In contrast with the large-area campaign, Palmes tubes were not only located on the façade of residential buildings but also on lampposts. As in the large-area campaign all sites were away from local sources other than traffic. We excluded measurements near hot spots such as traffic lights and bus stations. Tubes were mounted at 62 locations in Amsterdam; of these, 25 were placed in traffic-dominated areas, and 37 were placed in nontraffic areas. Monitoring took place continuously. We replaced the tubes every 28 days and analyzed as described by Palmes et al. (1976), which resulted in a full year of data.

All monitoring locations were geocoded using a national geographic information system (GIS) database (Kadaster, Apeldoorn, the Netherlands) that contained coordinates for all home addresses in the Netherlands. References for the geographic databases (including traffic and land-use data) used in this study can be found in Supplemental Material, Annex A (doi:10.1289/ehp.0901818).

### Traffic data

Geographic information on traffic flow was collected from all authorities responsible for traffic management in the study area. The national government is responsible for the freeways; the provinces for the highways, main connection routes, and other country roads in rural areas; and the municipalities for all other roads and streets. In the large study area, there were 93 sources of traffic data: the national department of traffic, 3 provinces, and 89 municipalities. All authorities provided data on traffic flow and traffic composition by road segment. For all freeways, data were obtained from continuous automated counters. For most other roads, traffic flow was estimated from yearly 2- to 4-week automated counts in combination with traffic models, most commonly OmniTRANS (Omnitrans International, Deventer, the Netherlands). Data were provided for 94.1% of the nationally managed roads, 58.2% of the provincially managed roads, and 48.1% of the municipally managed road lengths. Most authorities in the study area (national, provincial, and municipal) provided traffic data for the years 2004 (52% of the available road segments), 2005 (13%), or 2006 (31%). When no data for 2006 were available, data from the most recent previous year were used to estimate the expected 2006 traffic flow (Beelen et al. 2007). If no data were provided, quiet roads or small streets were assigned a minimal flow of 1,225 vehicles per 24 hr (Beelen et al. 2007), which was applied to none of the nationally managed roads and to 31.2% and 44.6% of the provincially and municipally managed road lengths, respectively. Altogether, traffic flow data was available for 87.3% of the total managed road lengths in the study area. Information on traffic composition was also available for 86.9%. These data were linked to a geo-database of all roads in the Netherlands. For each measurement site, we defined traffic flow in circular buffers (100 m and 250 m), distance to and traffic flow at the nearest road [distinguishing between the total (all) and heavy-duty traffic, such as trucks and buses] for different road types: all roads, busy roads (traffic load of > 5,000 vehicles/24 hr), main roads (load of > 10,000 vehicles/24 hr), and freeways. All distances to roads were log transformed *a priori* to allow for the nonlinear (exponential) decay of air pollution concentrations with distance to the road. All flow variables were categorized by distance (25, 50, 100, 250, and 500 m). All traffic variables used were derived using ArcGIS software (Version 9, ESRI, Redlands CA, USA).

### Land-use data

Information on land use in the large study area was derived from the European land-use database [Coordination of Information on the Environment (CORINE), European Environment Agency (EEA), Copenhagen, Denmark], available at a 100 × 100 m grid. For 10 different categories— residential, industry, transport, port, airport, waste or construction, urban green, forest, agriculture, and combined green space (urban green, forest, and agriculture)—we calculated the percentage of land use in circular buffers with radii of 300 m, 1 km, and 5 km around the monitoring sites. We adapted the resolution of the available data provided using the methodology described by Beelen et al. (2007, 2009); this process resulted in a total of 30 land-use variables.

For the city-specific model, the percentage of land use in 2006 from a 5 × 5 m grid map was calculated for circular buffers with radii of 25, 50, 100, 250, and 500 m. The land-use categories that were available in this detailed grid were railroad, road, freeway, building, business, industry, greenhouses, agriculture, urban green, forest, playground, sports ground, other tiled surfaces, water, combined green space (agriculture, urban green, forest, playground, and sports ground) and combined roads (road, highway, and freeway).

For the large-area and the city-specific LUR models, the number of inhabitants in circular buffers with radii of 100 m, 300 m, 1 km, and 5 km was calculated from the national population density database. The larger buffer sizes represent the potential impact of area level sources (e.g., all industrial or residential emissions) on measured concentrations, rather than the impact of a specific road or point source.

### Imputation of missing concentration data

In the large-area campaign, 10.6% of samplers were lost; for the city-specific campaign, 3.7% of the tubes were lost. Based on the available data, we imputed missing values 10 times using the MICE (multivariate imputation by chained equations) procedure in R (version 2.8.0; R Foundation for Statistical Computing, Vienna, Austria) and incorporated information on site type (rural, urban, or traffic). The differences between the 10 imputed data sets were small, as only a small percentage of the observations was missing. From each imputed data set, we calculated the mean concentration for each location to estimate the annual mean values.

As a result of the multiple imputations applied to the measurement data sets, 10 complete data sets for each of the two campaigns were available. We calculated model parameters by imputation and then combined these parameters using the SAS MIANALYZE procedure (version 9.1; SAS Institute Inc., Cary NC, USA) to account for the uncertainty about the imputed values.

### LUR model development and validation

The relationship between land-use and traffic variables and NO_2_ concentration at the measurement sites was studied by multiple linear regression analysis. We constructed regression models using a supervised forward-selection procedure (Beelen et al. 2009). We added variables to the regression model in four steps: traffic variables, traffic-related land-use variables, population density-related land-use variables, and other land-use variables (such as industry and green space).

In each of these steps, the variable with the highest *R*^2^ based on simple (or univariate) linear regression analysis was selected first. In selecting the best predictor, all categories (i.e., different buffer sizes) were tested separately, and only the best predictor per group (i.e., each land-use category) was selected for further testing; thus, no overlapping categories were included in the model. Variables with the second, third, etc. highest *R*^2^ were then added one by one and included in the multiple (or multivariate) regression model if the adjusted *R*^2^ improved by at least 1% and if the sign of each of the regression coefficients remained as expected.

Because of the larger and more diverse area, the regional background concentration calculated as the inverse distance weighted mean concentration of rural background measurement sites within a radius of 50 km (measurements made in the large-area campaign) was included *a priori* in the large-area model for all urban sites. For the rural background sites, the locally measured concentration was used as the local background concentration.

After all available variables had been tested, the resulting model was reexamined. We excluded variables with the highest *p*-values one at a time if the adjusted *R*^2^ remained mostly unchanged (difference in adjusted *R*^2^ < 1%). The reduced model was preferred.

The final model was evaluated using an internal leave-one-out cross-validation procedure (Hoek et al. 2008). We additionally evaluated the two models by comparing the concentrations predicted by one model for sites used to develop the other model. To study the additional value of the more complete traffic and land-use data, the large-area model was also developed using limited traffic data (without municipal road data) and the city-specific model was also developed using less-detailed land-use data (CORINE).

### Dispersion model

In this study, the Dutch modeling tool CAR (Eerens et al. 1993; Velders and Diederen 2009) was used because according to Dutch air quality regulations, this is the model that should be used in built-up areas of the Netherlands to calculate traffic-related air pollution. An extensive description of the model is available in Supplemental Material, Annex B (doi:10.1289/ehp.0901818). CAR is an empirical dispersion model derived from a more comprehensive Gaussian dispersion model (Vardoulakis et al. 2003). The model adds a local traffic contribution to a large-scale concentration map, which is updated every year. This large-scale concentration map is calculated from measurement data of the National Air Quality Monitoring Network in combination with the modeled contribution of important sources, such as industries, in the Netherlands and other European countries (see Supplemental Material, Annex B). Traffic contribution is calculated by multiplying the traffic emissions with a dispersion factor. Traffic emissions are calculated from traffic intensity, traffic composition, and default speed-dependent national emission factors. The dispersion factor depends on street configuration (buildings, trees), distance to the center of the road, and average annual wind speed (see Supplemental Material, Annex B). The CAR model can be applied to a maximum distance of 60 m from a road.

We used CAR (version 6.1.1; TNO, Utrecht, the Netherlands) to predict 2006 annual mean NO_2_ concentrations in this study for both sets of monitoring locations, using meteorology for the year 2006. The input information in the model includedexact geocoded location, traffic flow (vehicles/24 hr) and composition (percentage of cars, vans, trucks and buses), distance to the center of the road (meters), and categorical information on driving speed, road type, and the presence of trees.

### Comparison of LUR and dispersion models

Because the CAR atmospheric dispersion model is used to predict air pollution concentrations for almost all roads for which traffic information is available in the Netherlands, we compared concentrations observed at the measurement sites with the CAR predictions as well. Performance of the dispersion model was compared with the LUR models at the monitoring sites located in Amsterdam (13 monitoring sites of the large-area campaign and 62 monitoring sites of the city-specific campaign). This comparison was done by evaluating scatter plots and correlations between observed and predicted concentrations and between predictions by the different models.

## Results

### Large-area LUR model

Table 1 shows the distribution of the measured concentrations and the predictor variables for the large-area model. Table 2 shows the change in NO_2_ concentrations per interquartile range increase in the predictors in this model and the explained variance of this model (*R*^2^ = 87%). Internal leave-one-out cross-validation resulted in a full-model *R*^2^ of 84%. In Supplemental Material, Figure 2 (doi:10.1289/ehp.0901818), we show a plot of the predicted and observed concentrations.

We also investigated the performance of the large-area model for the Amsterdam subregion of the study area. The resulting *R*^2^ of 79% for these 13 sites was only slightly less than in the original model (internal cross-validated *R*^2^ = 84%) [see Supplemental Material, Figure 3 (doi:10.1289/ehp.0901818)]. When we excluded all 13 Amsterdam sites from the model, which left 47 sites including the city of Rotterdam, the model performance expressed as *R*^2^ was 87%.

To evaluate the added value of the more complete traffic data, we developed a model using traffic data for nationally and provincially managed roads only. This resulted in a model [see Supplemental Material, Figure 4 (doi:10.1289/ehp.0901818)] including three predictor variables: background concentration, percentage of land-use categories residential, and port, in a 5-km circular buffer. The estimated coefficients for background concentration and residential land use were similar to those of the model with more complete traffic data (data not shown). The explained variance (*R*^2^ = 73%), however, was substantially lower than for the original model (*R*^2^ = 87%).

### City-specific LUR model

Table 1 shows the distribution of the measured concentrations for the city-specific model. Additionally, concentrations ranged from 24.8 to 39.1 μg/m^3^ at urban background sites and from 42.2 to 75.1 μg/m^3^ at traffic sites. The change in NO_2_ concentrations per interquartile range increase in predictors for this model (*R*^2^ = 72%, leave-one-out cross-validated *R*^2^ = 65%) are shown in Table 3 [observed vs. predicted plot in Supplemental Material, Figure 2 (doi:10.1289/ehp.0901818)]. As shown by this figure, the model performs well for observed concentrations up to approximately 55 μg/m^3^. At higher concentrations, the model underestimates the NO_2_ concentration. A map of the predicted NO_2_ contours for all of Amsterdam is shown in Figure 5 of the Supplemental Material (doi:10.1289/ehp.0901818).

To evaluate the added value of high-resolution land-use data for this model, we developed a model using CORINE (EEA) land-use data instead of high-resolution land-use data. In the final model [Supplemental Material, Figure 4 (doi:10.1289/ehp.0901818)], the same two traffic variables (distance to the nearest main road and traffic flow at the nearest busy road within 50 m) and the percentage of land-use category “port” in a 5-km circular buffer were included. The explained variance (*R*^2^) of the city-specific model with lower-resolution land-use data was 69%, only slightly less than that of the original city-specific model (72%).

### LUR model evaluation by independent sets of measurements

**Figure 1:**
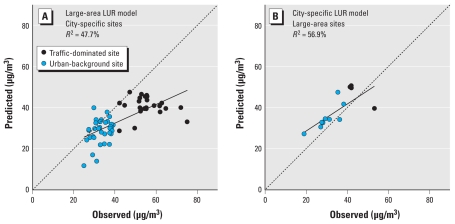
Evaluation of large-area and city-specific LUR models for measurements sites in Amsterdam, the Netherlands: predicted NO_2_ concentrations from one LUR-model versus observed concentrations at measurement sites that were used to develop the other LUR model. (*A*) Estimations by the large-area LUR, city-specific sites. (*B*) Estimations by the city-specific LUR, large-area sites. The dotted line indicates where observed equals predicted concentration.

Figure 1 shows plots of the observed NO_2_ concentrations at sites used to develop one LUR model and predicted concentrations from the other LUR model. Both LUR models performed less well in predicting NO_2_ concentrations at the sites that were used to develop the other model. Applying the large-area model to sites of the city-specific campaign (*n* = 62) (Figure 1A) resulted in an *R*^2^ of 48%, much lower than the *R*^2^ (72%) (Table 3) of the city-specific LUR for the sites of the city-specific campaign used to develop the model and the internal cross-validation *R*^2^. Applying the city-specific model to the Amsterdam sites of the large-area campaign resulted in an *R*^2^ of 57% (*n* = 13) (Figure 1B), much lower than the *R*^2^ of the large-area model for the Amsterdam sites of the large-area campaign (79%) [Supplemental Material, Figure 3 (doi:10.1289/ehp.0901818)] and the internal cross-validation *R*^2^.

### Dispersion model

Predictions from the CAR model were highly correlated with predictions from the two LUR models [Supplemental Material, Figure 6 (doi:10.1289/ehp.0901818)]. The agreement between CAR and both LUR models was higher for the 13 large-area campaign sites in Amsterdam (*R*^2^ = 89%) than for the 62 city-specific campaign sites (*R*^2^ = 75%).

**Figure 2:**
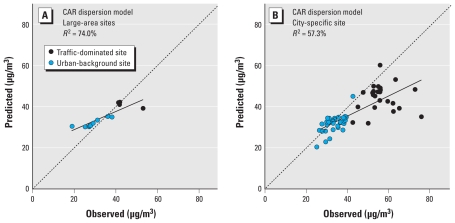
Observed and CAR dispersion model predicted NO_2_ concentrations at measurement sites in Amsterdam, the Netherlands. (*A*) CAR estimations for the large-area sites. (*B*) CAR estimations for the city-specific sites. The dotted line indicates where observed equals predicted concentration.

Figure 2 shows the CAR dispersion-model predictions and observed concentrations at the Amsterdam measurement sites of the large-area campaign (Figure 2A) and the sites of the city-specific campaign (Figure 2B). The CAR model predictions explain a large fraction of the variability in observed concentrations at the 13 Amsterdam sites of the large-area campaign (Figure 2A), but a systematic overestimation of background concentrations and underestimation of local traffic contributions to concentrations is evident. The CAR model explains a lower percentage of observed variability in concentrations at the city-specific sites (Figure 2B). As in the case of the city-specific LUR model, the dispersion model systematically underestimates the highest exposed traffic-dominated sites.

When we compared the percentage-explained variability (*R*^2^) of the LUR models at the independent monitoring sites, the CAR model performed slightly better than did the two LUR models. The percentage-explained variability at the city-specific sites was 57% for the CAR model (Figure 2B) and 48% for the large-area LUR model (Figure 1A). The percentage-explained variability at the large-area sites was 74% for the CAR model (Figure 2A) and 57% for the large-area LUR model (Figure 1B). However, when we accounted for the underestimation and overestimation, we concluded that the dispersion model did not perform better than the LUR models.

## Discussion

Two LUR models were developed for two independent sets of NO_2_ measurements. Both models explained a large percentage of the measured spatial variation (*R*^2^ for the large-area LUR = 87%; *R*^2^ for the city-specific LUR = 72%). Internal leave-one-out cross-validation *R*^2^s were only slightly lower (84% and 65%, respectively). Both LUR models performed less well in predicting concentrations at an independent set of monitoring sites than was expected from internal cross-validation (*R*^2^ large area = 48% vs. 84%; city specific = 57% vs. 65%). More complete traffic information improved the predictive power of the LUR models more than detailed land-use data. The dispersion model CAR did not perform better in predicting concentrations at independent monitoring sites than the two LUR models.

### Evaluation of LUR models

Two LUR models were developed that explained a high percentage of observed variability in measured NO_2_ concentrations. In internal leave-one-out cross-validations, percentages of explained variability were high as well, suggesting good applicability of the models to unmeasured locations. However, the models explained less variability when applied to the monitoring sites from the other sampling campaign. The main reason for this is probably because the sampling sites have been selected in different ways (see discussion below). As LUR models are generally developed to estimate ambient pollution levels at unmeasured locations in the study area (e.g., homes of study participants), the implication is that the sampling locations must be selected very carefully to reflect the type of location to which the model will be applied. If residential exposure assessment is the goal of LUR model development, measurements at the façade are probably a better choice than measurements at curbside.

The two measurement campaigns used in this study differed in year of monitoring (2006 vs. 2007), sampler (Palmes tube vs. Ogawa badge), temporal resolution (continuous vs. four 1-week samples), and site selection criteria (the large-area campaign was performed for the purpose of LUR modeling; the city-specific campaign consisted of selected locations from a routine monitoring program), which may have influenced cross-validation results. In previous LUR studies, both strategies (purpose designed and routine monitoring) to collect measurement data have been used regularly (e.g., Beelen et al. 2007; Briggs et al. 2000). However, the samplers in the city-specific campaign were often placed slightly closer to the road than in the large-area campaign. Although subtle, these systematic differences between measurement sites in both campaigns may explain, in part, the poorer predictions of the models for the sites used to develop the other model. Year of sampling may not have been important, as the correlation between concentrations measured in 2006 and 2007 at a subset of 35 sites from the city-specific campaign was 0.98. Continuous measurements performed at an urban background site of the national network in Amsterdam also showed similar concentrations during both measurement campaigns (32.0 and 32.2 μg/m^3^, respectively), indicating little (temporal) difference in NO_2_ levels between campaigns. As both samplers correlate highly with chemiluminescence monitors, differences between samplers are unlikely to be important. Several LUR studies have shown that spatial contrasts can be assessed with four 1- to 2-week sampling campaigns. However, absolute concentrations may deviate from annual mean concentrations (Hoek et al. 2008).

Few other studies have done out-of-sample validations of LUR models. In a study by Stedman et al. (1997), the model *R*^2^ was 97% (based on continuous NO_2_ monitors); in validation (using passive measurements at other locations), this dropped to 36%. Henderson et al. (2007), however, developed an LUR using passive measurements (model *R*^2^ = 56%), which scored higher (69%) in validation using continuous monitors.

The scale of the large-area model is somewhere between the metropolitan (e.g., Jerrett et al. 2007; Sahsuvaroglu et al. 2006) or national (e.g., Beelen et al. 2007; Stedman et al. 1997) scale of most other LUR models developed previously. The city-specific model, however, focuses on a metropolitan area. The availability of two LUR models for the same area provided the opportunity to compare the performance of LUR models originally developed for different geographic scales. The concentrations at traffic-dominated sites of the city-specific campaign, which were more often situated near complicated high-traffic situations, were largely underestimated by the large-area LUR model. Although hot-spot concentrations were still underestimated, application of the city-specific LUR model resulted in a better prediction with a much smaller mean residual of 2 μg/m^3^. Predictions of both models for urban background locations in both campaigns and traffic-dominated locations in the large-area campaign, however, were within the range of the measured concentrations.

### Value of detailed traffic and land-use information

In this study we put a large effort in gathering complete and detailed traffic information from all municipalities. Data from national and provincial authorities were readily available. Typically, most of the streets that people live by are municipal roads; therefore, traffic on these roads is important for exposure assessment used in epidemiological studies. Our effort resulted in participation of all municipalities, providing traffic data for 31% of the municipal roads. Traffic load could thus be assigned to 87% of the total road length in the study area. In a previous Dutch study (Beelen et al. 2007), 59% of the municipalities provided data, resulting in data for 14% of the municipal roads. Recalculation of the large-area model using limited traffic data (national and provincial only) resulted in a lower explained variance of that model (*R*^2^ = 73% vs. 87% for the recalculated and original large-area LUR, respectively) [Supplemental Material, Figures 2 and 4 (doi:10.1289/ehp.0901818)]. For other areas in which traffic is the main source of air pollution, the situation could be similar.

For Amsterdam, high-resolution land-use data were available, which is reflected by the higher information density shown on the city map. Smaller surfaces such as playgrounds or canals are not considered in a low-resolution map but can add up to an important part of the city surface area. Two of the high-resolution land-use variables (water and green space) were included in the city-specific LUR model. Recalculating the city-specific LUR model using land-use data at a lower resolution, however, showed that the added value of detailed land-use data in the model fit was limited (*R*^2^ = 69 vs. 72% for the recalculated and original city-specific LUR, respectively) [Supplemental Material, Figures 2 and 4 (doi:10.1289/ehp.0901818)]. When forced to prioritize in future studies, obtaining complete traffic data would therefore be preferred over obtaining higher resolution land-use data.

### Comparison of LUR models and a dispersion model

We conducted a comparison of the three approaches to model NO_2_ concentrations in Amsterdam. In the comparison, we found remarkable similarities between concentrations predicted by the large-area LUR and the dispersion model; the model predictions were highly correlated and showed very similar levels. Possible explanations are that the same traffic data and similar traffic predictors (traffic flow at the nearest road and a distance variable) were used in both models. Background concentration and residential land use together, as used in the large-area LUR model, seem to be equivalent to the large-scale concentration included in the dispersion model. Measurements used to estimate background levels in the LUR model and to calibrate the large-scale concentrations in the dispersion model were done independently, thus not causing similarities. The restriction of the dispersion model to the estimation of concentrations at a distance of no more than 60 m from a road (Eerens et al. 1993) may explain the differences between the dispersion and the city-specific LUR model.

The fit of the CAR dispersion model seems better for the 13 Amsterdam sites of the large-area campaign than for the sites in the city-specific campaign (Figure 2). Differences in the campaigns discussed above may have contributed to this finding. Differences in monitoring year and temporal resolution are unlikely explanations. These factors would have resulted in better agreement for the city-specific sites, as CAR predictions were made for the year 2006 for both data sets. Possible explanations include the smaller fraction of traffic sites among the large-area sampling sites (traffic sites are more difficult to model) and the range in concentrations. As in the case of the application of the large-area LUR model to city-specific sites and previous LUR studies (Briggs et al. 1997, 2000; Rosenlund et al. 2008; Ross et al. 2006; Sahsuvaroglu et al. 2006), the dispersion model was unable to predict the highest (hot-spot) concentrations observed in the city-specific campaign well. Additional evaluations of the locations with the highest concentrations in the city-specific campaign showed that most of these locations are situated near complicated high traffic situations such as congested busy roads. From these data, it is hard to conclude which model is most appropriate for estimating concentrations in Amsterdam, as most of the measurement data available were used in developing the city-specific model.

The few other studies comparing dispersion and LUR models have typically found that LUR models perform at least as well as the dispersion models considered (Vardoulakis et al. 2003). The comparison, however, depends on the particular model and its ability to model small-scale variations. The CAR model is a semiempirical model derived from a more detailed Gaussian model and adapted to calculate air quality near roads (Vardoulakis et al. 2003). The model is able to model small-scale variations in urban areas, but is not optimal for modeling dispersion along highways, so our results may not be generalizable to near-highway applications.

## Conclusion

A large-area LUR and a city-specific LUR model, developed for two independent sets of NO_2_ measurements, explain a large percentage of the measured spatial variation. Both LUR models performed less well than results found from internal leave-one-out cross-validation, possibly related to differences in site selection. Evaluation of the value of using high-resolution data showed that more complete traffic information adds much more to the model fit of LUR models than detailed land-use data. The dispersion-model CAR did not predict concentrations at independent monitoring sites better than the two LUR models.

## Figures and Tables

**Table 1:** Distribution of observed average NO_2_ concentrations and predictor variables used in the large-area (Northwest Netherlands) and city-specific (Amsterdam) multivariate LUR models.

Model, concentration/predictor	Median	Range
Large-area LUR model (*n* = 60)		
Measured NO_2_ concentration^a^ (μg/m^3^)	25.1	10.5–53.1
Regional background concentration (μg/m^3^)	20.7	10.8–25.4
Traffic volume at nearest road (vehicles/24 hr)	1,225	195.4–37132.8
Distance to nearest busy road^b^ (m)	103.4	0–1409.8
Residential land use in a 5-km buffer (%)	28.5	0.8–63.9

City-specific LUR model (*n* = 62)		
Measured NO_2_ concentration^a^ (μg/m^3^)	37.9	24.8–75.1
Traffic volume at nearest busy road b within 50 m (vehicles/24 hr)	0	0–29640.2
Distance to nearest main road^c^ (m)	113.5	9.1–2845.1
Green space in a 250-m buffer (%)	27.5	0.5–76.3
Water in a 100-m buffer (%)	4.9	0–30.8

a NO_2_ concentrations: average of 10 imputed data sets.

b ≥ 5,000 vehicles/24 hr.

c ≥ 10,000 vehicles/24 hr.

**Table 2:** Change in NO_2_ concentrations per interquartile range increase in predictor variables used in the large-area multivariate LUR model (*R*^2^ = 87%, adj*R*^2^ = 85%; cross-validation *R*^2^ = 84%, adj*R*^2^ = 82%).

Large-area LUR	Estimate^a^	SE^a^	*p*-Value
Intercept	10.7	3.9	0.008
Background concentration (μg/m^3^)	3.4	0.8	< 0.0001
Traffic volume at nearest road (vehicles/24 hr)	1.2	0.3	< 0.0001
Distance to nearest busy road^b^ (m)	−4.0	1.2	0.002
Residential land use in a 5-km buffer (%)	6.1	1.1	< 0.0001

Adj, adjusted.

a Per interquartile range. Background concentration = 4.4 μg/m^3^; traffic volume = 2,668 vehicles/24 hr; distance = 110 m; residential land use = 26%.

b ≥ 5,000 motor vehicles per 24 hr.

**Table 3:** Change in NO_2_ concentrations per interquartile range increase in predictor variables used in the city-specific multivariate LUR model (*R*^2^ = 72%, adj*R*^2^ = 69%; cross-validation *R*^2^ = 65%, adj*R*^2^ = 63%).

City-specific LUR	Estimate^a^	SE^a^	*p*-Value
Intercept	56.2	5.5	< 0.0001
Traffic volume at nearest busy road^b^ within 50 m (vehicles/24 hr)	7.1	2.3	0.003
Distance to nearest main road^c^ (m)	−7.6	2.6	0.005
Green space in a 250-m buffer (%)	−4.6	1.6	0.005
Water in a 100-m buffer (%)	2.7	1.5	0.076

Adj, adjusted.

a Per interquartile range. Traffic volume = 14,052 vehicles/24 hr; distance = 249 m; green space = 26%; water = 13%.

b ≥ 5,000 vehicles/24 hr.

c ≥ 10,000 vehicles/24 hr.
